# Electrospinning Nanofiber Mats with Magnetite Nanoparticles Using Various Needle-Based Techniques

**DOI:** 10.3390/polym14030533

**Published:** 2022-01-28

**Authors:** Al Mamun, Lilia Sabantina, Michaela Klöcker, Alexander Heide, Tomasz Blachowicz, Andrea Ehrmann

**Affiliations:** 1 Junior Research Group “Nanomaterials”, Faculty of Engineering and Mathematics, Bielefeld University of Applied Sciences, 33619 Bielefeld, Germany; al.mamun@fh-bielefeld.de (A.M.); lilia.sabantina@fh-bielefeld.de (L.S.); 2 Faculty of Engineering and Mathematics, Bielefeld University of Applied Sciences, 33619 Bielefeld, Germany; michaela.kloecker@fh-bielefeld.de (M.K.); alexander.heide@fh-bielefeld.de (A.H.); 3 Institute of Physics—Center for Science and Education, Silesian University of Technology, 44-100 Gliwice, Poland; tomasz.blachowicz@polsl.pl

**Keywords:** freestanding nanofiber mats, magnetic nanoparticles, needle-based electrospinning, coaxial spinning, dynamic mechanical analysis (DMA), atomic force microscopy (AFM), scanning electron microscopy (SEM)

## Abstract

Electrospinning can be used to produce nanofiber mats containing diverse nanoparticles for various purposes. Magnetic nanoparticles, such as magnetite (Fe_3_O_4_), can be introduced to produce magnetic nanofiber mats, e.g., for hyperthermia applications, but also for basic research of diluted magnetic systems. As the number of nanoparticles increases, however, the morphology and the mechanical properties of the nanofiber mats decrease, so that freestanding composite nanofiber mats with a high content of nanoparticles are hard to produce. Here we report on poly (acrylonitrile) (PAN) composite nanofiber mats, electrospun by a needle-based system, containing 50 wt% magnetite nanoparticles overall or in the shell of core–shell fibers, collected on a flat or a rotating collector. While the first nanofiber mats show an irregular morphology, the latter are quite regular and contain straight fibers without many beads or agglomerations. Scanning electron microscopy (SEM) and atomic force microscopy (AFM) reveal agglomerations around the pure composite nanofibers and even, round core–shell fibers, the latter showing slightly increased fiber diameters. Energy dispersive X-ray spectroscopy (EDS) shows a regular distribution of the embedded magnetic nanoparticles. Dynamic mechanical analysis (DMA) reveals that mechanical properties are reduced as compared to nanofiber mats with smaller amounts of magnetic nanoparticles, but mats with 50 wt% magnetite are still freestanding.

## 1. Introduction

Electrospinning enables the production of nanofibers or nanofiber mats from diverse polymers or polymer blends [1,2,3]. Additional materials, such as ceramics, metals, or metal oxides, can be embedded in the form of nanoparticles [4,5,6]. Even functionalization with molecules is possible [7,8]. Depending on the material composition, such nanofiber mats can be used for various applications, such as biotechnology, biomedicine and tissue engineering [9,10,11], filters for fluids and gases [12,13,14], and energy harvesting and storage [15,16,17]. Most recent applications can be found in water purification, H_2_ production, environmental protection [18,19,20,21], or as catalysts [22,23,24].

Among the broad range of nanoparticular materials, magnetic nanoparticles can be applied to diverse purposes, either practically for electromagnetic shielding, hyperthermia therapy or as catalysts [25,26,27], or as a model system for basic research on magnetic properties of nanofibers and nanofiber composites [28,29,30].

In all these cases, the distribution of the magnetic nanoparticles and their amount are highly relevant. In many cases, homogeneously distributed nanoparticles are favored [31,32,33]. The nanoparticle distribution is especially relevant, since it defines the magnetic properties of the nanofiber mats. Single magnetic nanoparticles, or nanoparticles in sufficient distance to their neighbors, often show magnetic properties different from the bulk material or larger agglomerations of nanoparticles. Bulk magnetic materials typically have domain walls, and magnetization reversal is usually performed by domain wall nucleation and propagation [34,35]. Small nanoparticles often consist of only one domain, enabling coherent rotation of the magnetization and thus a completely different magnetization reversal process, resulting in different coercive fields and potentially different remanence [36,37]. The sizes of such single-domain nanoparticles differ, depending on the material and respective magnetic properties, but also on the nanoparticles’ shapes [36,37]. Single nanoparticles, in which the magnetization can rotate freely, become superparamagnetic, i.e., the hysteresis loop is closed, and the coercive field is zero [38,39]. Both these effects, however, are strongly affected by agglomerations of nanoparticles inside a matrix, e.g., in a nanofiber [40]. Investigating the nanoparticle distribution inside a nanofiber mat is thus highly important for estimating its magnetic properties.

In contrast, for most applications, a high amount of magnetic nanoparticles inside the polymeric matrix is favorable. A previous study revealed that poly(acrylonitrile) (PAN) nanofiber mats including magnetite or nickel ferrite nanoparticles (25 wt% in the spinning solution), electrospun with a needleless machine “Nanospider Lab,” resulted in the formation of large beads in which the magnetic nanoparticles agglomerated [41]. Here, we report on nanofiber mats containing 50 wt% magnetite nanoparticles in the spinning solution, electrospun with a needle-based machine, as either common fibers or core–shell fibers, on a flat or a rotating collector. Our results show that, while the nanofiber mats spun with a common needle contain large irregularities and the fibers are mostly deformed, the core–shell fibers and the corresponding nanofiber mats show a highly regular morphology without beads or large agglomerations.

## 2. Materials and Methods

### 2.1. Electrospinning

The electrospinning solution was produced by dissolving 13 wt% PAN (X-PAN, Dralon, Dormagen, Germany) in dimethyl sulfoxide (DMSO, min 99.9%, S3 Chemicals, Bad Oeynhausen, Germany), which was then mixed with a magnetic stirrer at room temperature for 1 h. Magnetite nanoparticles (Fe_3_O_4_, particle size 50–100 nm, Merck KGaA, Darmstadt, Germany) of 50 wt% of the previous spinning solution were added to the solution by manual stirring, followed by ultrasonic treatment at 35 °C and a frequency of 37 kHz for 30 min. The amount of magnetite in the overall spinning solution was thus 33.3 wt%. As a reference, a PAN/magnetite spinning solution containing 20 wt% Fe_3_O_4_ as well as pure PAN nanofiber mats from 16% PAN in DMSO were prepared.

Electrospinning was performed by a needle-based electrospinning system (Spinbox, from Bioinicia, Paterna, Valencia, Spain), applying a voltage of max. 18 kV along a tip-collector distance of 20 cm. Besides a flat collector, a rotating collector (diameter 100 mm, 300–400 rpm) was used. The flow rate through the needle with an inner diameter of 0.6 mm was set to 10 µL/min for spinning with one solution. A coaxial needle with an inner diameter of 0.6 mm and an outer ring diameter of 0.5 mm was used to produce core–shell fibers, with flow rates of 10 µL/min for the core (13 wt% PAN) and 10 µL/min for the shell (13 wt% PAN + 50 wt% magnetite). Spinning was performed at a chamber temperature of 20–23 °C and a relative humidity of 27–34%. The samples prepared are named MF (PAN/magnetite, flat collector), MR (PAN/magnetite, rotating collector), CSF (core–shell, flat collector), CSR (core–shell, rotating collector), and R (reference, pure PAN), respectively.

### 2.2. Characterization

The samples’ morphology was investigated by a confocal laser scanning microscope (CLSM) VK-8710 (Keyence) for large-area scans, a Zeiss Sigma 300 VP scanning electron microscope (SEM) and a FlexAFM Axiom (Nanosurf, Liestal, Switzerland) in tapping mode, using Tap190Al-G (CSR and CSF samples) and Multi 75M-G (MF and MR samples) tips. The nanoparticle distribution was measured by energy dispersive X-ray spectroscopy (EDS) with a Quantax 70 EDX unit (Bruker Nano GmbH, Berlin, Germany), attached to an SEM Hitachi TM-3000 (Hitachi High-Technologies Corporation, Tokyo, Japan). Nanofiber diameters were measured in SEM micrographs by ImageJ (version 1.53e, 2021, National Institutes of Health, Bethesda, MD, USA), using 100 fibers per sample.

An Excalibur 3100 (Varian, Inc., Palo Alto, CA, USA) with a spectral range of 4000 cm^−1^ to 700 cm^−1^ was used for chemical investigation by Fourier transform infrared (FTIR) spectroscopy. Data were averaged over 32 scans and corrected for atmospheric noise.

Dynamic mechanical analysis (DMA) was performed by a Q800 (TA Instruments, Eschborn, Germany), applying a preload force of 0.001 N, followed by a force-ramp of 0.05 N/min until break. Measurements were performed at 23 °C, using a sample width of 5.3 mm. While mechanical investigations of nanofibrous composites are often complicated due to the constraints to clamp components [42], the nanofiber mats under investigation in this study could unambiguously be measured in this way.

## 3. Results and Discussion

**Figure 1:**
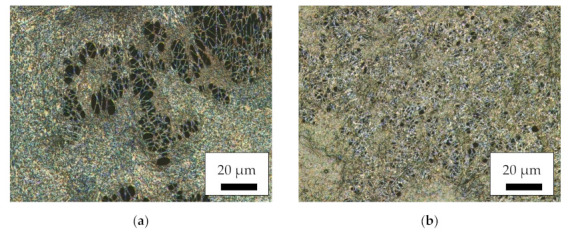

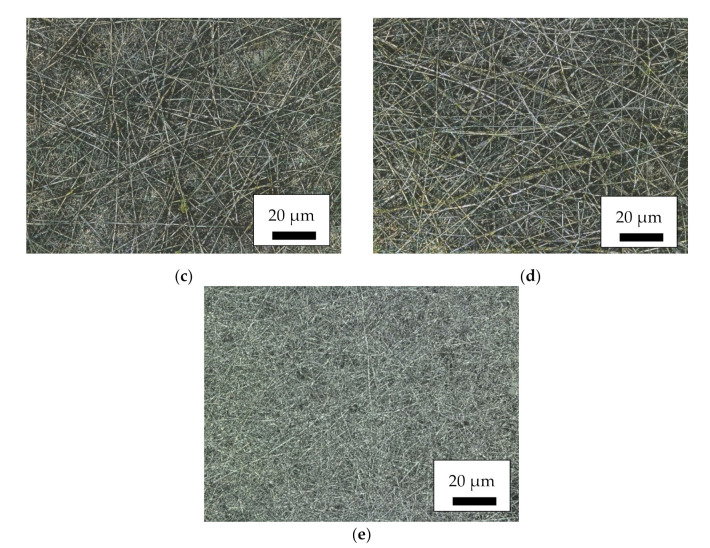
Confocal laser scanning microscope (CLSM) images of the magnetic nanofiber mats: (**a**) MF; (**b**) MR; (**c**) CSF; (**d**) CSR; (**e**) pure PAN as a reference.

For an overview of the produced nanofiber mats with a relatively large field of view, Figure 1 depicts CLSM images of the different magnetic samples as well as of a pure PAN nanofiber mat for comparison. Here, it is clearly visible that the MF and MR samples have a much more distorted and irregular morphology than the core–shell fiber samples CSF and CSR. The latter, nevertheless, have much larger fiber diameters than the pure PAN nanofiber mat. No large difference is visible between samples electrospun on the flat and on the rotating collector. Apparently, the interaction between high voltage, tip-collector distance and rotational speed of the collector is not sufficient for aligning the fibers, as it is often reported in the literature for high rotation speeds [43,44,45]. However, fiber alignment was not the aim of this study and is thus not further optimized.

**Figure 2:**
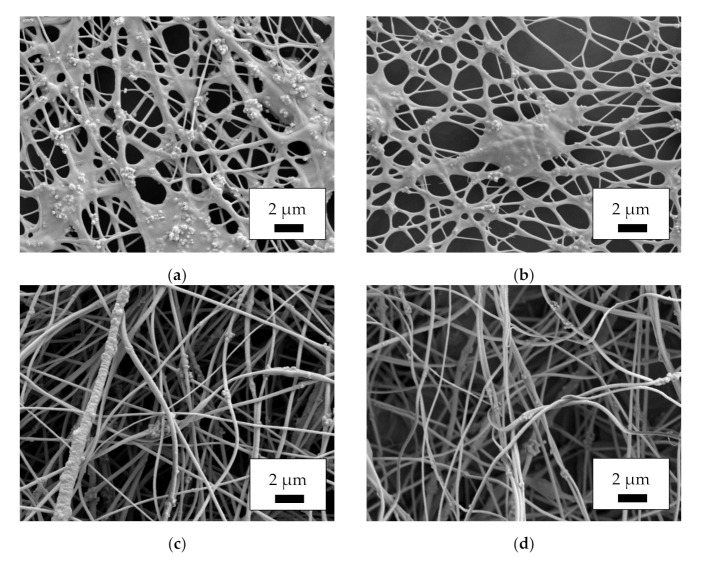
Scanning electron microscope (SEM) images of the magnetic nanofiber mats, taken with acceleration voltage 12 kV and SE detector after sputtering with 20 nm gold: (**a**) MF; (**b**) MR; (**c**) CSF; (**d**) CSR.

Proceeding from mat morphology to fiber morphology, Figure 2 depicts SEM images, taken at a magnification of 5000×. As could already be estimated from Figure 1, the single fibers in the MF and MR samples do not show the desired nanofiber morphology, but are highly agglomerated, with nanoparticles protruding from the areas between the fibers. Similar agglomerations are often seen in nanofiber mats containing magnetic nanoparticles [40,41]. One possible explanation for this effect is that the spinning solution was not homogeneous enough to enable the formation of perfect fibers. Conversely, it must be taken into account that a large amount of magnetite nanoparticles, as applied here, will significantly alter the viscosity, conductivity and surface tension of a spinning solution, so that even in case of perfectly distributed nanoparticles, the preparation of unaltered nanofibers cannot be expected.

The images of the core–shell fibers CSF and CSR, however, show the desired round nanofibers with only few agglomerations. While only the shells of these nanofibers contain a large number of magnetic nanoparticles, i.e., overall, there is a smaller amount of magnetic material per fiber length than in the MF and MR samples’ fibers, the magnetic properties of such nanofibers are more dependent on the distribution of the nanoparticles than on the overall amount [40], making these fibers relevant for various applications.

**Figure 3:**
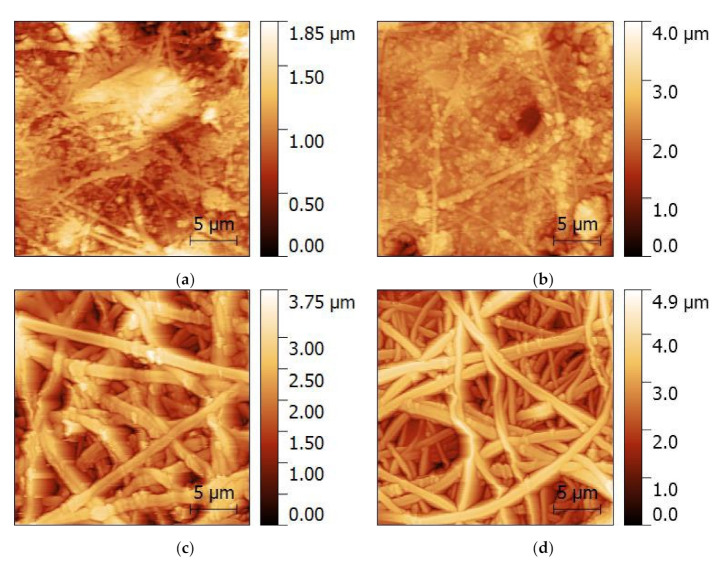
Atomic force microscopy (AFM) images of the magnetic nanofiber mats: (**a**) MF; (**b**) MR; (**c**) CSF; (**d**) CSR.

A look onto the nanofiber surfaces with even higher resolution is made possible by AFM, as depicted in Figure 3. Again, the MF and MR samples show significant deviations from the desired nanofiber morphology. Agglomerations of nanoparticles are visible along the fibers and between them. The core–shell fibers, in contrast, show only few nanoparticles protruding from the fibers again, underlining that these nanofiber mats have the desired morphology with only very few deviations from perfectly even, round nanofibers, as they can be produced with smaller amounts of magnetic nanoparticles [46]. The root mean square (RMS) values, giving an idea of the surface roughness, are (237 ± 110) nm (MF), (311 ± 80) nm (MR), (523 ± 82) nm (CSF), and (686 ± 62) nm (CSR), respectively. These values indicate that the nanofiber mats from core–shell fibers (CSF and CSR) are “rougher” than the simple MF and MR fibers, which is consistent with the observation in Figure 3 that there are no large pores in the MF and MR nanofiber mats, while the separated nanofibers in CSF and CSR surround large, deep pores in which the next fibers are much lower.

**Figure 4:**
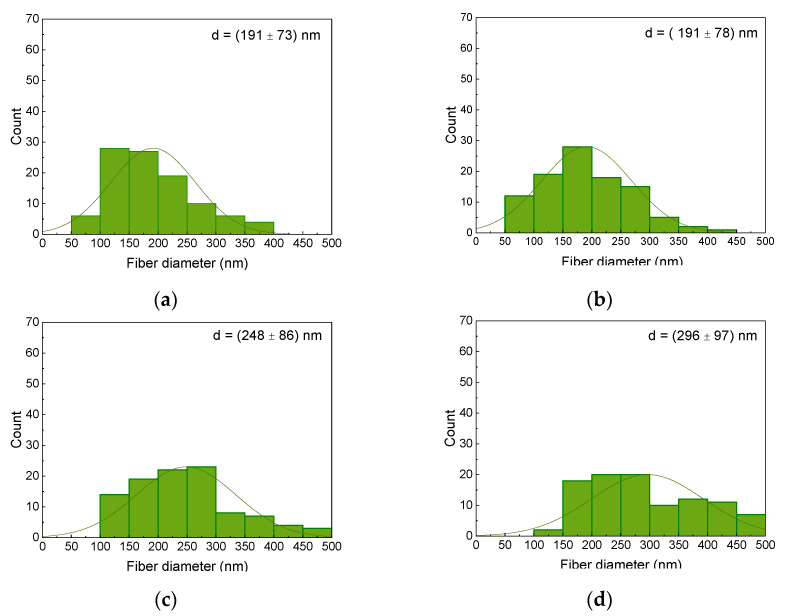
Diameter distributions of the magnetic nanofiber mats: (**a**) MF; (**b**) MR; (**c**) CSF; (**d**) CSR.

Taking into account the pure fibers without agglomerations, the AFM images show that the core–shell fibers have a larger diameter than the fibers of the MF and MR samples. The corresponding fiber diameter distributions are depicted in Figure 4, showing indeed a tendency towards thicker fibers for the coaxial spinning process. It should be mentioned that the fiber diameters are generally much larger than those of previously produced nanofibers from spinning solutions containing 20 wt% magnetite or nickel ferrite, with average diameters of approx. 100 nm for both magnetic nanoparticles [46].

**Figure 5:**
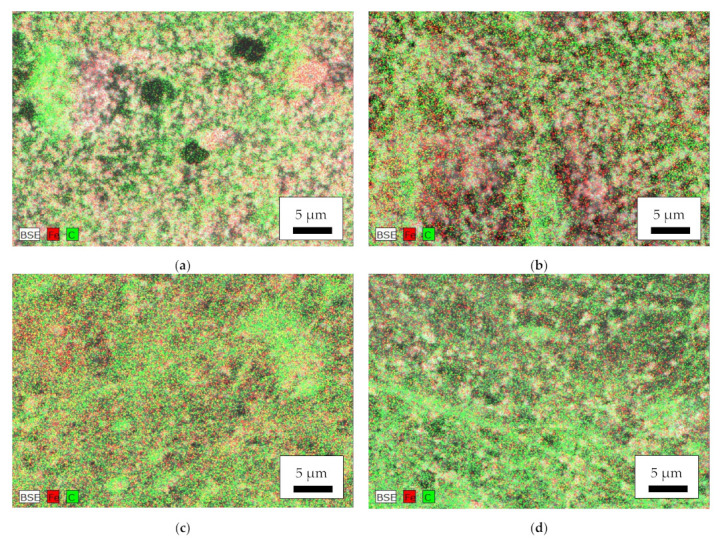
Energy dispersive X-ray spectroscopy (EDS) of the magnetic nanofiber mats: (**a**) MF; (**b**) MR; (**c**) CSF; (**d**) CSR. BSE = backscattered electrons, Fe = iron, C = carbon.

Aside from their morphology, the nanoparticles’ distribution is of utmost importance. Figure 5 thus depicts EDS maps of the magnetic samples. While the amount of carbon, indicating the polymeric part of the nanofiber mats, shows some local variations in the nanofibrous structure and the beads and agglomerations, especially visible in the MR sample (Figure 5b), the iron, indicating the magnetite nanoparticles, is well distributed, with no significant agglomerations visible. This is contrary to [41] where agglomerations in the beads and smaller amounts of magnetic nanoparticles in the fibers were found.

**Figure 6:**
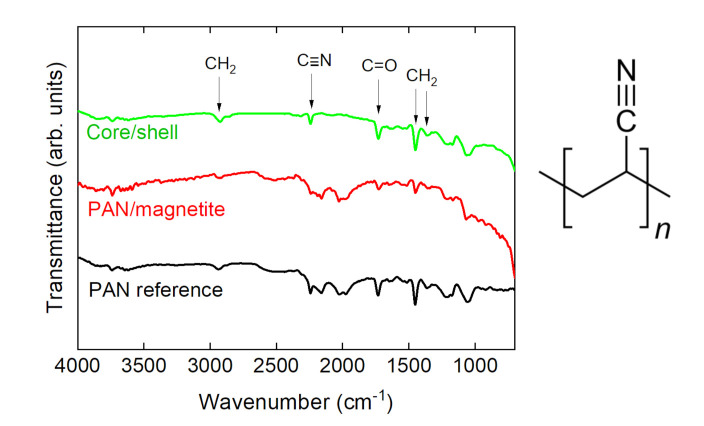
Fourier transform infrared (FTIR) spectra of exemplarily chosen nanofiber mats and molecular structure of PAN.

The chemical investigation by FTIR revealed no unexpected properties of the PAN/magnetite nanofiber mats. Figure 6 depicts the typical peaks of PAN, i.e., CH_2_ bending and stretching vibrations at 2938 cm^−1^, 1452 cm^−1^, and 1380 cm^−1^, the stretching vibrations of the nitrile group at 2240 cm^−1^, and the carbonyl stretching peak at 1731 cm^−1^ [47]. It should be mentioned that metals generally cause deviations from a flat baseline, as visible here for small wavenumbers, while the artifact around 2100 cm^−1^ stems from the incompletely compensated absorption of the diamond ATR crystal. As expected, no differences were visible between nanofiber mats spun on the flat and the rotating collector.

**Figure 7:**
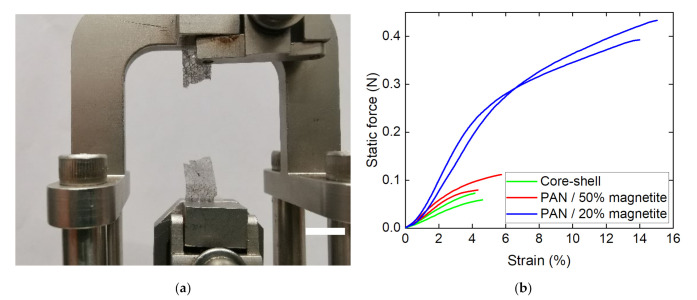
Dynamic mechanical analysis (DMA) of the nanofiber mats under investigation: (**a**) CSR sample after breaking; (**b**) force–strain curves of different samples, grouped as PAN with 20% magnetite (blue lines), MF and MR PAN/magnetite samples (red lines) and CSF and CSR core–shell fiber samples (green lines), respectively.

Finally, DMA measurements were performed, comparing MF and MR PAN/magnetite nanofiber mats with the CSF and CSR core–shell samples as well as with a composite nanofiber mat out of 16% PAN and only 20% magnetite. Figure 7 depicts the measurement principle and exemplary results.

While the smaller amount of only 20% magnetite results in a clear increase in forces—as could be expected, since fewer nanoparticles disturbed the fiber continuity—the nanofiber mats composed of core–shell fibers unexpectedly showed slightly smaller forces at break than the MF and MR PAN/50% magnetite samples. This can be attributed to the large agglomerations of nanoparticles/polymer composite material between the fibers (cf. Figure 2a,b), which reduced the fiber quality on the one hand, but resulted in a better connection between the existing fibers on the other. Most important, however, is the fact that all nanofiber mats investigated in this study were freestanding, i.e., could be separated from the substrates unambiguously. This finding shows that coaxial electrospinning, in particular, allows for producing fibers with a large number of magnetic nanoparticles, which are stable enough to be used as freestanding parts of batteries or other applications.

## 4. Conclusions

PAN/magnetite nanofiber mats were electrospun as composite fibers and as core–shell fibers with a PAN core and PAN/magnetite shell. While the first show strongly altered morphology of fibers and mats, the core–shell fibers are straight and round as pure PAN nanofibers. All nanofibers with magnetic nanoparticles have larger diameters than pure PAN nanofibers. The magnetic nanoparticles were evenly distributed in the nanofiber mats. DMA tests showed that the large number of magnetic nanoparticles reduced the mechanical properties of the nanofibers containing 50 wt% magnetite in the shell or in the whole fiber, but all magnetic nanofiber mats were still freestanding, allowing for their use as freestanding electrodes in applications such as batteries.

## Data Availability

All data gained during this study are reported in the paper.
